# A Single Vitamin D_3_ Bolus Supplementation Improves Vitamin D Status and Reduces Proinflammatory Cytokines in Healthy Females

**DOI:** 10.3390/nu14193963

**Published:** 2022-09-24

**Authors:** Hadeil M. Alsufiani, Shareefa A. AlGhamdi, Huda F. AlShaibi, Sawsan O. Khoja, Safa F. Saif, Carsten Carlberg

**Affiliations:** 1 Department of Biochemistry, Faculty of Sciences, King Abdulaziz University, Jeddah 21589, Saudi Arabia; 2 Vitamin D Pharmacogenomics Research Group, King Abdulaziz University, Jeddah 21589, Saudi Arabia; 3 Experimental Biochemistry Unit, King Fahd Medical Research Center, King Abdulaziz University, Jeddah 21589, Saudi Arabia; 4 Embryonic Stem Cell Unit, King Fahd Medical Research Center, King Abdulaziz University, Jeddah 21589, Saudi Arabia; 5 Institute of Animal Reproduction and Food Research, Polish Academy of Sciences, PL 10-748 Olsztyn, Poland; 6 Institute of Biomedicine, School of Medicine, University of Eastern Finland, FI 70211 Kuopio, Finland

**Keywords:** vitamin D deficiency, single high dose, vitamin D_3_ supplementation, proinflammatory cytokines, IL6, IL8, TNF, 25(OH)D_3_

## Abstract

Vitamin D deficiency is a global health problem that not only leads to metabolic bone disease but also to many other illnesses, most of which are associated with chronic inflammation. Thus, our aim was to investigate the safety and effectiveness of a single high dose of vitamin D_3_ (80,000 IU) on vitamin D status and proinflammatory cytokines such as interleukin (IL)6, IL8 and tumor necrosis factor (TNF) in healthy Saudi females. Fifty healthy females were recruited and orally supplemented with a single vitamin D_3_ bolus (80,000 IU). All participants donated fasting blood samples at baseline, one day and thirty days after supplementation. Serum 25-hydroxyvitamin D_3_ (25(OH)D_3_), IL6, IL8, TNF, calcium, phosphate, parathyroid hormone (PTH) and blood lipid levels were determined. Serum 25(OH)D_3_ significantly increased one and thirty days after supplementation when compared with baseline without causing elevation in calcium or phosphate or a decrease in PTH to abnormal levels. In contrast, the concentrations of the three representative proinflammatory cytokines decreased gradually until the end of the study period. In conclusion, a single high dose (80,000 IU) is effective in improving serum vitamin D status and reducing the concentration of the proinflammatory cytokines in a rapid and safe way in healthy females.

## 1. Introduction

Vitamin D_3_ is a micronutrient that can be synthesized in human skin from the cholesterol precursor 7-dehydrocholesterol through energy provided by the ultraviolet-B (UVB) component of sunlight [1]. Recent lifestyle and work–life changes towards indoor activities as well as the use of clothing and sunscreen for sunburn protection outdoors have reduced the chances of filling up vitamin D_3_ stores. This results in far lower average vitamin D status in today’s modern societies than in more traditionally living populations [2,3,4,5]. Even in sunny Saudi Arabia, a substantial proportion of the population is considered vitamin D-deficient [6]. This increases the risk not only of muscle weakness (sarcopenia) and early onset of osteoporosis but also leads to an increase in autoimmune diseases, such as type 1 diabetes, arthritis, multiple sclerosis, cancer, cardiovascular diseases and Alzheimer’s disease [7,8]. Therefore, vitamin D deficiency is a global health problem that not only leads to musculoskeletal problems but also to many other illnesses, most of which are associated with chronic inflammation [9,10].

In the liver, vitamin D_3_ is hydroxylated to 25(OH)D_3_, which is the most stable vitamin D_3_ metabolite circulating in the blood. Therefore, 25(OH)D_3_ serum levels serve as a biomarker for the vitamin D status. In the kidneys, 25(OH)D_3_ is further metabolized to the physiologically most active vitamin D metabolite, 1,25-dihydroxyvitamin D_3_ (1,25(OH)_2_D_3_) [11]. The lipophilic nature of 1,25(OH)_2_D_3_ allows the molecule to pass through cellular and nuclear membranes and to act in the nucleus as a high-affinity ligand to the transcription factor vitamin D receptor (VDR), i.e., 1,25(OH)_2_D_3_ has a direct effect on gene regulation [12,13]. Besides the kidneys, 1,25(OH)_2_D_3_ is also synthesized locally in a number of tissues and cell types expressing VDR. Taking all presently investigated tissues and cell types together, there are more than 20,000 VDR binding sites in the human genome, and significant changes in the transcriptome profile occur in over 1000 human genes [14].

Examples of vitamin D target tissues include immune cells such as T cells, B cells and monocytes, which are the major components of peripheral blood mononuclear cells (PBMCs) [15,16,17]. One hallmark of vitamin D’s effects is the regulation of genes involved in the regulation of inflammatory processes. Accordingly, there is an interplay between vitamin D signaling and other signaling cascades involved in inflammation [18,19].

The impact of 1,25(OH)_2_D_3_ treatment on the expression of the proinflammatory cytokines IL6, IL8 and TNF was extensively studied in PBMCs from healthy donors, primary monocytes/macrophages as well as in monocytic cell lines, indicating that the VDR ligand causes their down-regulation on an mRNA and protein level [20,21,22,23,24,25,26,27]. Importantly, not only does the treatment of cell culture models with 1,25(OH)_2_D_3_ promote changes in gene expression, but also the supplementation of individuals with vitamin D_3_ leads to the same results. Most of these studies were conducted on patients with diverse inflammatory diseases, such as COVID-19, colorectal cancer, irritable bowel syndrome, obesity and diabetes [28,29,30,31,32,33,34], and only a few studies were performed with healthy individuals [35,36,37].

Vitamin D intervention studies usually use different doses of vitamin D_3_ supplementation either daily or weekly for several weeks or months, and only a few of them used a single high dose. The pharmacology of vitamin D shows that the proper half-life for dose periods is longer than daily supplementation, and many dosing regimens suggest that high vitamin D_3_ doses at less frequent periods are more suitable and have become a broad practice [38]. Moreover, from an experimental point of view, the use of a single high vitamin D_3_ dose is more suitable for observing the direct effects of vitamin D on the expression of its target genes, such as multiple cytokines, both on the mRNA and protein level. Accordingly, the aim of this study was to investigate the safety and effectiveness of a single high dose of vitamin D_3_ (80,000 IU) on the vitamin D status and the serum levels of representative proinflammatory cytokines IL6, IL8 and TNF in healthy Saudi females.

## 2. Materials and Methods

### 2.1. Study Design and Participants

**Figure 1:**
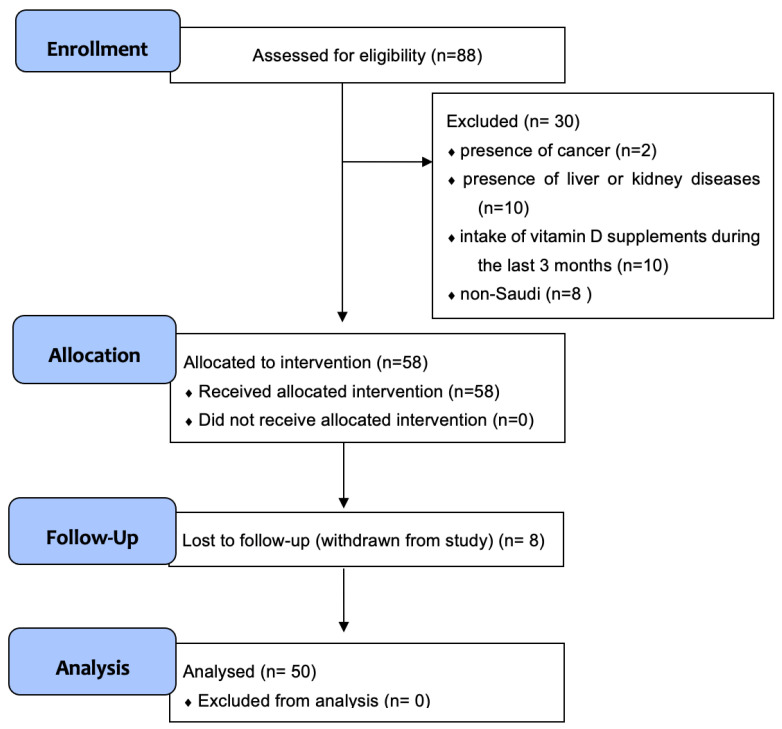
Flow chart showing the flow of the participants throughout the study. n = number of individuals.

Fifty healthy Saudi females aged between 18 and 60 were recruited from King Abdul Aziz University and King Fahad Medical Research Center’s staff and their families from January to December 2019. The total sample size was calculated based on a power analysis (using G*Power software, version 3.1.9.7, Düsseldorf, Germany) that indicated a 95% chance of a 0.5 effect size between the tested groups at the 5% level (two-tailed). Exclusion criteria included the presence of cancer, liver or kidney diseases, the intake of vitamin D supplements during the last three months, and non-Saudis. All participants received a single high dose of vitamin D_3_ (80,000 IU) orally administered (Figure 1). This dose was chosen since previous experience in the vitamin D intervention studies VitDbol (https://clinicaltrials.gov/ct2/show/NCT02063334) (accessed on 19 September 2022) and VitDHiD (https://clinicaltrials.gov/ct2/show/NCT03537027) (accessed on 19 September 2022) indicated that 80,000 IU vitamin D_3_ is a safe monthly dose in healthy individuals. This study was approved by the ethical committee of the Faculty of Medicine, King Abdulaziz University (reference number 30-18), and all participants provided written informed consent.

### 2.2. Anthropometric Measurements

Height and weight were measured by using an electronic scale and a portable stadiometer from Seca (Hamburg, Germany), respectively, and the body mass index (BMI) was calculated for all participants. In addition, waist and hip circumference were measured using Seca tape, and the waist-to-hip ratio (WHR) was then calculated.

### 2.3. Biochemical Measurements

All participants donated fasting blood samples at three different time points; at baseline (day 0), after one day (day 1) and after thirty days (day 30) of oral administration of a single high dose of vitamin D_3_ (80,000 IU). Serum was isolated and stored at −80 °C for later measurements of biochemical parameters including lipid profile, phosphorus (PHOS), calcium (CAL), parathyroid hormone (PTH), 25(OH)D_3_ and proinflammatory cytokines.

Quantitative determination of serum cholesterol (CHOL), low-density lipoproteins (LDL) and triglycerides (TAG) was performed using a Siemens Dimension Vista instrument. Serum CAL and PHOS were measured using a kit from Siemens Healthcare Diagnostic Limited, Dimension Vista System UK (Cat. No K1023 and Cat. No K1061, respectively). Serum PTH was measured using a chemiluminescent microparticle immunoassay (CMIA) technique kit from Abbott (Cat. No 8K25). Serum vitamin D status was determined by measuring 25(OH)D_3_ via the Abbott Architect 25-OH Vitamin D assay kit. Finally, the proinflammatory cytokines IL6, IL8 and TNF were measured using Human Interleukin 6 ELISA Kit by Bioassay Technology Laboratory (Cat. No E0089Hu), Human Interleukin 8 ELISA Kit by Bioassay Technology Laboratory (Cat. No E0089Hu) and Human Tumor Necrosis Factor Alpha ELISA Kit by Bioassay Technology Laboratory (Cat. No E0082Hu), respectively.

### 2.4. Statistical Analysis

All statistical analyses were performed using IBM SPSS software version 24 (SPSS Inc., Chicago, IL, USA) and graphs were represented using GraphPad prism 7. Data were presented as mean ± standard error of mean (SEM). Repeated measures one-way analysis of variance (ANOVA) followed by Bonferroni’s multiple comparison test was used to determine the significant differences in mean serum levels of 25(OH)D_3_, IL6, IL8 and TNF, CHOL, TAG, LDL, PHOS, CAL and PTH between days 0, 1 and 30 of vitamin D_3_ supplementation. The statistical significance threshold was taken as *p* < 0.05.

## 3. Results

Fifty females with a mean age of 29 years participated in this study. At baseline, their mean BMI was 23.6 kg/m^2^ and their mean WHR was 0.77. All biochemical parameters including CHOL, LDL, TAG, PHOS, CAL, and PTH were in the normal range intervals indicating a good health status of all participants (Table 1). After supplementation with vitamin D_3_, no changes were found in most biochemical parameters except in CHOL, PHOS and PTH levels. The changes in these parameters were minor and did not reach abnormal levels.

**Figure 2:**
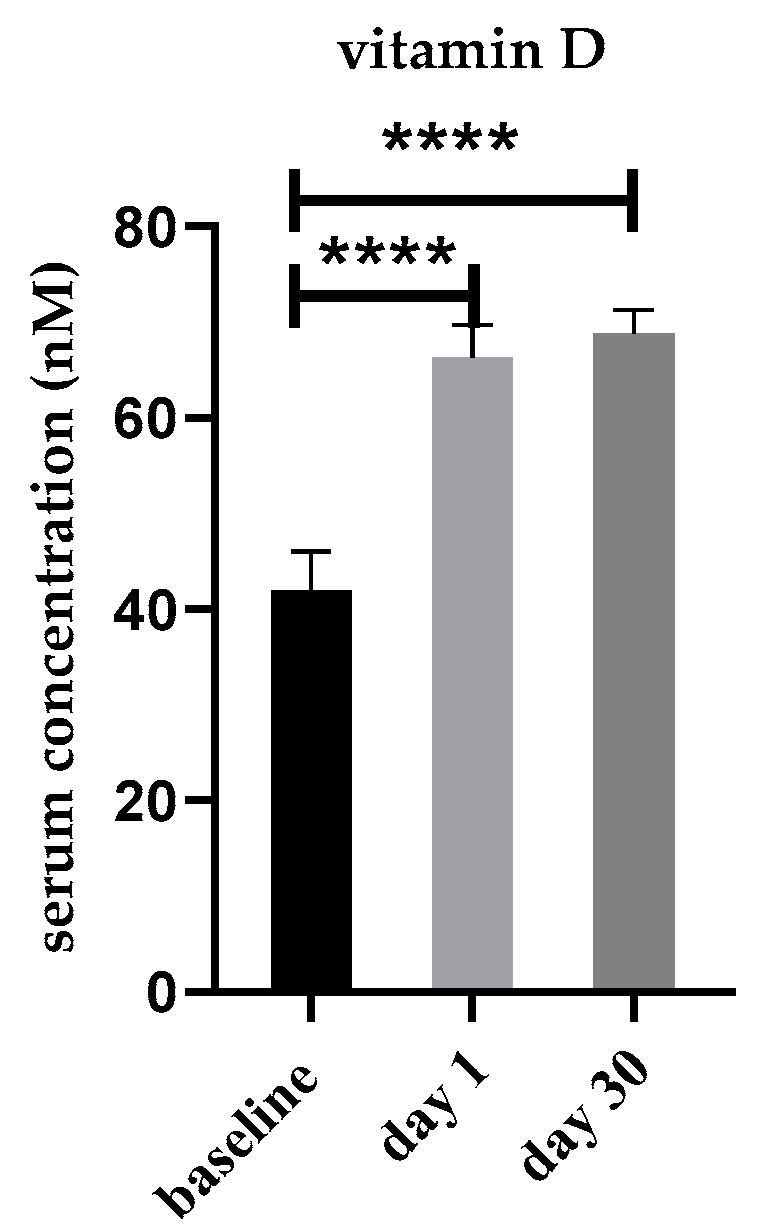
Mean serum 25(OH)D_3_ concentrations at baseline, day 1 and day 30 following a single high dose of vitamin D_3_ supplementation (n = 50). Error bars show SEM. **** *p* < 0.0001.

The mean serum 25(OH)D_3_ concentration at baseline was 41.9 ± 4.1 nM, and 72% of study participants had an insufficient vitamin D status of less than 50 nM (Table 2). The average vitamin D status significantly increased to 66.3 ± 3.5 nM at day 1 and 68.9 ± 2.5 nM at day 30 (Figure 2). This represents an average increase by 24.4 and 26.9 nM and a shift from deficiency and insufficiency to sufficiency for 76% and 94% of the study participants, respectively, at days 1 and 30 after vitamin D_3_ bolus supplementation (Table 2).

**Figure 3:**
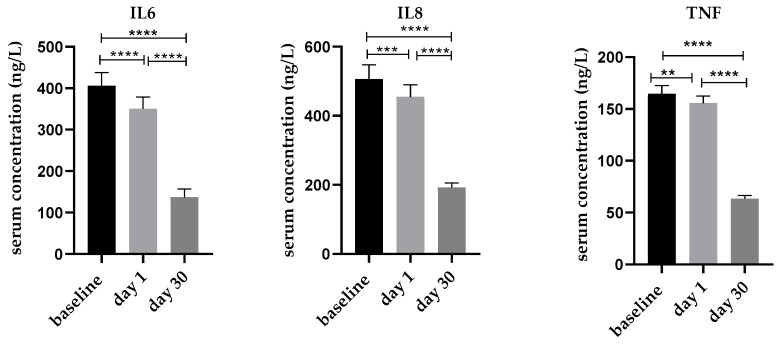
Mean serum levels of the proinflammatory cytokines IL6, IL8 and TNF at baseline, day 1 and day 30 following a single high dose of vitamin D_3_ supplementation (n = 50). Error bars show SEM. ** *p* < 0.01, *** *p* < 0.001, **** *p* < 0.0001.

Mean serum IL6 concentrations significantly decreased from 405 ± 30 ng/L at baseline to 350 ± 30 ng/L at day 1 and even 137 ± 20 ng/L at day 30 (Figure 3). This represents an average decrease by 55 and 269 ng/L, respectively. Similar trends were also found for serum IL8 concentrations, where baseline levels gradually decreased from 506 ± 40 ng/L to 455 ± 35 ng/L at day 1 and 192 ± 10 ng/L at day 30 (Figure 3) and for serum TNF levels, which significantly decreased from 165 ± 8 ng/L at baseline to 156 ± 7 ng/L at day 1 and 63 ± 3 ng/L at day 30 (Figure 3). Interestingly, neither Pearson nor Spearman correlation analysis provided any significant correlation between the vitamin D status and the expression level of the proinflammatory cytokines.

## 4. Discussion

The purpose of this study was to investigate the effectiveness of a single high dose of vitamin D_3_ (80,000 IU) on the vitamin D status and the representative proinflammatory cytokines IL6, IL8 and TNF in healthy Saudi females. The vitamin D_3_ bolus increased the vitamin D status within a month by nearly 27 nM and achieved a shift in the study participants from vitamin D deficiency and insufficiency to sufficiency. In fact, the approximately 60% increase in vitamin D status was already visible within one day. This result is comparable to a previous study conducted in female adults supplemented with a single high dose of vitamin D_3_ (100,000 IU) [39]. For comparison, when a lower dose was used (50,000 IU), the percent increase in serum 25(OH)D_3_ concentrations was only 30% [40]. Other previous studies conducted on adults supplemented daily with different doses of vitamin D_3_ ranging from 200 to 600 IU for 2 to 5 months showed a similar or lower percent increase in serum 25(OH)D_3_ levels [41,42,43,44].

A potential chronic toxicity of vitamin D would result from the administration of doses far above the maximally recommended daily dose of 4000 IU vitamin D_3_ for months or years that will increase serum 25(OH)D_3_ concentrations to 250 nM or more. In addition to elevated serum 25(OH)D_3_ concentrations, vitamin D toxicity can be diagnosed by severe hypercalcemia and by very low or undetectable PTH activity [45]. Accordingly, oral supplementation with a single high dose (80,000 IU) is sufficient to increase the level of serum 25(OH)D_3_ in a rapid, suitable and safe way, as none of our study participants reached a vitamin D status of more than 125 nM. Moreover, no abnormal changes were found in either serum calcium or PTH levels after supplementation.

An association between high serum 25(OH)D_3_ concentrations and low concentrations of the proinflammatory cytokines IL6, IL8 and TNF was reported previously [46,47,48]. In the present study, low serum 25(OH)D_3_ concentrations at baseline were observed in concordance with the high concentration of the proinflammatory cytokines, but these correlations did not reach statistical significance. Importantly, a single vitamin D_3_ bolus was sufficient to significantly increase the vitamin D status within one month and in parallel resulted in the reduction in protein levels of IL6, IL8 and TNF by 67, 62 and 61%, respectively, at the end of the study. The downregulation of the expression of the proinflammatory cytokines may be explained by the increased activation of VDR by an elevated vitamin D status. The latter may have caused a raise in 1,25(OH)_2_D_3_ levels in the nuclei of VDR-expressing PBMCs. Although the genes *IL6* and *TNF* are not known as primary vitamin D target genes, a network of secondary and indirect effects of VDR activation can lead to changes in their expression [49]. However, the *IL8* gene is known as a primary vitamin D target [50].

In contrast to our results, Smith et al. (2017) reported that a single high dose of vitamin D_3_ (250,000 IU) did not change serum IL6 and IL8 levels in healthy adults, which could be due to the small sample size of their study [36]. Moreover, daily supplementation with low doses of vitamin D_3_ (4000 IU) did not affect serum IL6 concentrations in healthy adults [35]. However, serum TNF concentrations were reported to decrease after supplementation of healthy male and female adults with 4000 IU vitamin D_3_ for 20 days [37]. Studies conducted on patients with inflammation-related diseases showed that daily supplementation with different doses of vitamin D_3_ ranging from 1000 to 50,000 IU for several weeks or months decreased not only serum TNF concentrations but also serum IL6 and IL8 levels [28,29,30,31,32,51,52,53,54,55]. Thus, a single high dose (80,000 IU) of vitamin D_3_ is as effective in reducing proinflammatory cytokines as daily doses.

## 5. Conclusions

An important finding of this study was that oral supplementation with a single high dose (80,000 IU) is effective in improving the serum’s vitamin D status and decreasing the concentration of the proinflammatory cytokines in a rapid, suitable and safe way in healthy females. This will help in preventing and reducing vitamin D deficiency, as well as related inflammatory diseases, in the general population. Further research needs to be performed in order investigate the effectiveness of this single high dose on pro- and anti-inflammatory markers in various inflammatory diseases.

## Figures and Tables

**Table 1:** Demographic and clinical characteristics of study participants at baseline, day 1 and day 30 following a single high dose of vitamin D_3_ supplementation (n = 50).

	Baseline	Day 1	Day 30
Age (years)	28.9 ± 0.9		
Height (cm)	158.9 ± 0.7	NA	NA
Weight (kg)	59.9 ± 1.8	NA	NA
BMI (kg/m^2^)	23.6 ± 0.7	NA	NA
Waist circumference (cm)	74.5 ± 2.1	NA	NA
Hip circumference (cm)	97.7 ± 2.5	NA	NA
WHR	0.77 ± 0.02	NA	NA
CHOL (mM)	4.34 ± 0.12	4.26 ± 0.12	4.13 ± 0.11 *
TAG (mM)	1.05 ± 0.07	2.39 ± 1.34	1.07 ± 0.08
LDL (mM)	2.78 ± 0.11	2.34 ± 0.18	2.53 ± 0.09
PHOS (mM)	1.24 ± 0.03	1.18 ± 0.03 *	1.24 ± 0.03
CAL (mM)	2.29 ± 0.02	2.24 ± 0.01	2.22 ± 0.02
PTH (pM)	5.25 ± 0.44	4.44 ± 0.30 *	4.17 ± 0.28 **

Data are presented as mean ± SEM. * *p* < 0.05, ** *p* < 0.01 when compared with baseline. NA: Data are not available.

**Table 2:** Prevalence of vitamin D deficiency among study participants at baseline, day 1 and day 30 following a single high dose of vitamin D_3_ supplementation (n = 50).

Serum Vitamin D Status *	Baseline N (%)	Day 1 N (%)	Day 30 N (%)
Deficiency 25(OH)D_3_ < 30 nM	24 (48%)	0 (0%)	0 (0%)
Insufficiency 25(OH)D_3_ of 30–50 nM	12 (24%)	12 (24%)	3 (6%)
Sufficiency 25(OH)D_3_ > 50 nM	14 (28%)	38 (76%)	47 (94%)

* classification was based on US Institute of Medicine (IOM).

## Data Availability

Data are available when requested.

## References

[1] Wacker M., Holick M.F. (2013). Sunlight and Vitamin D: A global perspective for health. Dermatoendocrinol.

[2] Carlberg C., Litwack G. (2016). Chapter Ten—Molecular approaches for optimizing vitamin D supplementation. Vitamins & Hormones.

[3] Carlberg C., Haq A. (2018). The concept of the personal vitamin D response index. J. Steroid Biochem. Mol. Biol..

[4] Luxwolda M.F., Kuipers R.S., Kema I.P., van der Veer E., Dijck-Brouwer D.A., Muskiet F.A. (2013). Vitamin D status indicators in indigenous populations in East Africa. Eur. J. Nutr..

[5] Luxwolda M.F., Kuipers R.S., Kema I.P., Dijck-Brouwer D.A., Muskiet F.A. (2012). Traditionally living populations in East Africa have a mean serum 25-hydroxyvitamin D concentration of 115 nmol/l. Br. J. Nutr..

[6] Al-Alyani H., Al-Turki H.A., Al-Essa O.N., Alani F.M., Sadat-Ali M. (2018). Vitamin D deficiency in Saudi Arabians: A reality or simply hype: A meta-analysis (2008–2015). J. Family Community Med..

[7] Holick M.F. (2007). Vitamin D deficiency. N. Engl. J. Med..

[8] Grant W.B., Boucher B.J., Al Anouti F., Pilz S. (2022). Comparing the evidence from observational studies and randomized controlled trials for nonskeletal health effects of vitamin D. Nutrients.

[9] Chun R.F., Liu P.T., Modlin R.L., Adams J.S., Hewison M. (2014). Impact of vitamin D on immune function: Lessons learned from genome-wide analysis. Front. Physiol..

[10] Di Filippo L., De Lorenzo R., Giustina A., Rovere-Querini P., Conte C. (2022). Vitamin D in osteosarcopenic obesity. Nutrients.

[11] Carlberg C., Polly P. (1998). Gene regulation by vitamin D_3_. Crit. Rev. Eukaryot. Gene Expr..

[12] Haussler M.R., Haussler C.A., Jurutka P.W., Thompson P.D., Hsieh J.C., Remus L.S., Selznick S.H., Whitfield G.K. (1997). The vitamin D hormone and its nuclear receptor: Molecular actions and disease states. J. Endocrinol..

[13] Carlberg C., Dunlop T.W. (2006). An integrated biological approach to nuclear receptor signaling in physiological control and disease. Crit. Rev. Eukaryot. Gene Expr..

[14] Tuoresmäki P., Väisänen S., Neme A., Heikkinen S., Carlberg C. (2014). Patterns of genome-wide VDR locations. PLoS ONE.

[15] Seuter S., Neme A., Carlberg C. (2016). Epigenome-wide effects of vitamin D and their impact on the transcriptome of human monocytes involve CTCF. Nucleic Acids Res..

[16] Ramagopalan S.V., Heger A., Berlanga A.J., Maugeri N.J., Lincoln M.R., Burrell A., Handunnetthi L., Handel A.E., Disanto G., Orton S.M. (2010). A ChIP-seq defined genome-wide map of vitamin D receptor binding: Associations with disease and evolution. Genome Res..

[17] Handel A.E., Sandve G.K., Disanto G., Berlanga-Taylor A.J., Gallone G., Hanwell H., Drabløs F., Giovannoni G., Ebers G.C., Ramagopalan S.V. (2013). Vitamin D receptor ChIP-seq in primary CD4+ cells: Relationship to serum 25-hydroxyvitamin D levels and autoimmune disease. BMC Med..

[18] Wöbke T.K., Sorg B.L., Steinhilber D. (2014). Vitamin D in inflammatory diseases. Front. Physiol..

[19] Malmberg H.R., Hanel A., Taipale M., Heikkinen S., Carlberg C. (2021). Vitamin D treatment sequence is critical for transcriptome modulation of immune challenged primary human cells. Front. Immunol..

[20] Zhang Y., Leung D.Y., Richers B.N., Liu Y., Remigio L.K., Riches D.W., Goleva E. (2012). Vitamin D inhibits monocyte/macrophage proinflammatory cytokine production by targeting MAPK phosphatase-1. J. Immunol..

[21] Willheim M., Thien R., Schrattbauer K., Bajna E., Holub M., Gruber R., Baier K., Pietschmann P., Reinisch W., Scheiner O. (1999). Regulatory effects of 1α,25-dihydroxyvitamin D_3_ on the cytokine production of human peripheral blood lymphocytes. J. Clin. Endocrinol. Metab..

[22] Joshi S., Pantalena L.C., Liu X.K., Gaffen S.L., Liu H., Rohowsky-Kochan C., Ichiyama K., Yoshimura A., Steinman L., Christakos S. (2011). 1,25-dihydroxyvitamin D_3_ ameliorates Th17 autoimmunity via transcriptional modulation of interleukin-17A. Mol. Cell Biol..

[23] Di Rosa M., Malaguarnera G., De Gregorio C., Palumbo M., Nunnari G., Malaguarnera L. (2012). Immuno-modulatory effects of vitamin D3 in human monocyte and macrophages. Cell Immunol..

[24] Müller K., Bendtzen K. (1992). Inhibition of human T lymphocyte proliferation and cytokine production by 1,25-dihydroxyvitamin D_3_. Differential effects on CD45RA+ and CD45R0+ cells. Autoimmunity.

[25] Panichi V., De Pietro S., Andreini B., Bianchi A.M., Migliori M., Taccola D., Giovannini L., Tetta C., Palla R. (1998). Calcitriol modulates in vivo and in vitro cytokine production: A role for intracellular calcium. Kidney Int..

[26] Rausch-Fan X., Leutmezer F., Willheim M., Spittler A., Bohle B., Ebner C., Jensen-Jarolim E., Boltz-Nitulescu G. (2002). Regulation of cytokine production in human peripheral blood mononuclear cells and allergen-specific th cell clones by 1α,25-dihydroxyvitamin D_3_. Int. Arch. Allergy Immunol..

[27] Prabhu Anand S., Selvaraj P., Narayanan P.R. (2009). Effect of 1,25 dihydroxyvitamin D_3_ on intracellular IFN-gamma and TNF-α positive T cell subsets in pulmonary tuberculosis. Cytokine.

[28] Sabico S., Enani M.A., Sheshah E., Aljohani N.J., Aldisi D.A., Alotaibi N.H., Alshingetti N., Alomar S.Y., Alnaami A.M., Amer O.E. (2021). Effects of a 2-week 5000 IU versus 1000 IU vitamin D_3_ supplementation on recovery of symptoms in patients with mild to moderate Covid-19: A randomized clinical trial. Nutrients.

[29] Haidari F., Abiri B., Iravani M., Ahmadi-Angali K., Vafa M. (2020). Randomized study of the effect of vitamin D and omega-3 fatty acids cosupplementation as adjuvant chemotherapy on inflammation and nutritional status in colorectal cancer patients. J. Diet. Suppl..

[30] Khalighi Sikaroudi M., Mokhtare M., Janani L., Faghihi Kashani A.H., Masoodi M., Agah S., Abbaspour N., Dehnad A., Shidfar F. (2020). Vitamin D_3_ supplementation in diarrhea-predominant irritable bowel syndrome patients: The effects on symptoms improvement, serum corticotropin-releasing hormone, and interleukin-6—a randomized clinical trial. Complement. Med. Res..

[31] Mirzaei K., Hossein-Nezhad A., Keshavarz S.A., Eshaghi S.M., Koohdani F., Saboor-Yaraghi A.A., Hosseini S., Tootee A., Djalali M. (2014). Insulin resistance via modification of PGC1α function identifying a possible preventive role of vitamin D analogues in chronic inflammatory state of obesity. A double blind clinical trial study. Minerva Med..

[32] Imanparast F., Javaheri J., Kamankesh F., Rafiei F., Salehi A., Mollaaliakbari Z., Rezaei F., Rahimi A., Abbasi E. (2020). The effects of chromium and vitamin D_3_ co-supplementation on insulin resistance and tumor necrosis factor-alpha in type 2 diabetes: A randomized placebo-controlled trial. Appl. Physiol. Nutr. Metab..

[33] Wimalawansa S.J. (2022). Rapidly increasing serum 25(OH)D boosts the immune system, against infections-sepsis and COVID-19. Nutrients.

[34] Hopefl R., Ben-Eltriki M., Deb S. (2022). Association between vitamin D levels and inflammatory markers in COVID-19 patients: A meta-analysis of observational studies. J. Pharm. Pharm. Sci..

[35] Chandler P.D., Scott J.B., Drake B.F., Ng K., Manson J.E., Rifai N., Chan A.T., Bennett G.G., Hollis B.W., Giovannucci E.L. (2014). Impact of vitamin D supplementation on inflammatory markers in African Americans: Results of a four-arm, randomized, placebo-controlled trial. Cancer Prev. Res..

[36] Smith E.M., Alvarez J.A., Kearns M.D., Hao L., Sloan J.H., Konrad R.J., Ziegler T.R., Zughaier S.M., Tangpricha V. (2017). High-dose vitamin D_3_ reduces circulating hepcidin concentrations: A pilot, randomized, double-blind, placebo-controlled trial in healthy adults. Clin. Nutr..

[37] Alkhedaide A.Q.H., Alshehri Z.S., Soliman M.M., Althumali K.W., Abu-Elzahab H.S., Baiomy A.A.A. (2016). Vitamin D_3_ supplementation improves immune and inflammatory response in vitamin D deficient adults in Taif, Saudi Arabia. Biomed. Res..

[38] Vieth R., Feldman D., Pike J.W., Adams J.S. (2011). Chapter 57—The pharmacology of vitamin D. Vitamin D.

[39] Witham M.D., Adams F., Kabir G., Kennedy G., Belch J.J., Khan F. (2013). Effect of short-term vitamin D supplementation on markers of vascular health in South Asian women living in the UK—A randomised controlled trial. Atherosclerosis.

[40] Raimundo F.V., Lang M.A., Scopel L., Marcondes N.A., Araújo M.G., Faulhaber G.A., Furlanetto T.W. (2015). Effect of fat on serum 25-hydroxyvitamin D levels after a single oral dose of vitamin D in young healthy adults: A double-blind randomized placebo-controlled study. Eur. J. Nutr..

[41] Yao P., Lu L., Hu Y., Liu G., Chen X., Sun L., Ye X., Zheng H., Chen Y., Hu F.B. (2016). A dose-response study of vitamin D_3_ supplementation in healthy Chinese: A 5-arm randomized, placebo-controlled trial. Eur. J. Nutr..

[42] Pilz S., Hahn A., Schön C., Wilhelm M., Obeid R. (2017). Effect of two different multimicronutrient supplements on vitamin D status in women of childbearing age: A randomized trial. Nutrients.

[43] Shirvani A., Kalajian T.A., Song A., Allen R., Charoenngam N., Lewanczuk R., Holick M.F. (2020). Variable genomic and metabolomic responses to varying doses of vitamin D supplementation. Anticancer Res..

[44] Pettersen J.A. (2017). Does high dose vitamin D supplementation enhance cognition?: A randomized trial in healthy adults. Exp. Gerontol..

[45] Marcinowska-Suchowierska E., Kupisz-Urbańska M., Łukaszkiewicz J., Płudowski P., Jones G. (2018). Vitamin D toxicity-a clinical perspective. Front. Endocrinol..

[46] De Vita F., Lauretani F., Bauer J., Bautmans I., Shardell M., Cherubini A., Bondi G., Zuliani G., Bandinelli S., Pedrazzoni M. (2014). Relationship between vitamin D and inflammatory markers in older individuals. Age.

[47] Laird E., McNulty H., Ward M., Hoey L., McSorley E., Wallace J.M., Carson E., Molloy A.M., Healy M., Casey M.C. (2014). Vitamin D deficiency is associated with inflammation in older Irish adults. J. Clin. Endocrinol. Metab..

[48] Liefaard M.C., Ligthart S., Vitezova A., Hofman A., Uitterlinden A.G., Kiefte-de Jong J.C., Franco O.H., Zillikens M.C., Dehghan A. (2015). Vitamin D and C-reactive protein: A Mendelian randomization study. PLoS ONE.

[49] Hanel A., Carlberg C. (2022). Time-resolved gene expression analysis monitors the regulation of inflammatory mediators and attenuation of adaptive immune response by vitamin D. Int. J. Mol. Sci..

[50] Ryynänen J., Carlberg C. (2013). Primary 1,25-dihydroxyvitamin D_3_ response of the interleukin 8 gene cluster in human monocyte- and macrophage-like cells. PLoS ONE.

[51] Ghorbani Z., Togha M., Rafiee P., Ahmadi Z.S., Rasekh Magham R., Djalali M., Shahemi S., Martami F., Zareei M., Razeghi Jahromi S. (2020). Vitamin D_3_ might improve headache characteristics and protect against inflammation in migraine: A randomized clinical trial. Neurol. Sci..

[52] Esfandiari A., Pourghassem Gargari B., Noshad H., Sarbakhsh P., Mobasseri M., Barzegari M., Arzhang P. (2019). The effects of vitamin D_3_ supplementation on some metabolic and inflammatory markers in diabetic nephropathy patients with marginal status of vitamin D: A randomized double blind placebo controlled clinical trial. Diabetes Metab. Syndr..

[53] Gagnon C., Daly R.M., Carpentier A., Lu Z.X., Shore-Lorenti C., Sikaris K., Jean S., Ebeling P.R. (2014). Effects of combined calcium and vitamin D supplementation on insulin secretion, insulin sensitivity and β-cell function in multi-ethnic vitamin D-deficient adults at risk for type 2 diabetes: A pilot randomized, placebo-controlled trial. PLoS ONE.

[54] Beilfuss J., Berg V., Sneve M., Jorde R., Kamycheva E. (2012). Effects of a 1-year supplementation with cholecalciferol on interleukin-6, tumor necrosis factor-alpha and insulin resistance in overweight and obese subjects. Cytokine.

[55] Pincikova T., Paquin-Proulx D., Sandberg J.K., Flodström-Tullberg M., Hjelte L. (2017). Clinical impact of vitamin D treatment in cystic fibrosis: A pilot randomized, controlled trial. Eur. J. Clin. Nutr..

